# Changes in Bronchiolitis Incidence During the Last Two Decades in Tampere, Finland: A Retrospective Study

**DOI:** 10.1097/INF.0000000000003662

**Published:** 2022-07-28

**Authors:** Tytti Vihikangas, Sauli Palmu, Anna-Maija Koivisto, Paula Heikkilä

**Affiliations:** From the * Tampere University, Faculty of Medicine and Health Technology; † Tampere Centre for Child, Adolescent and Maternal Health Research, Faculty of Medicine and Health Technology, Tampere University and Tampere University Hospital, Tampere, Finland; ‡ Tampere University, Faculty of Social Sciences

**Keywords:** Bronchiolitis, incidence, infant, epidemiology, respiratory syncytial virus

## Abstract

Bronchiolitis, a lower respiratory tract infection, causes a remarkable number of hospitalizations globally. The epidemiology follows the same pattern as respiratory syncytial virus (RSV), the most common pathogen in bronchiolitis. Epidemics have typically followed a biannual pattern in Nordic countries—first, a small epidemic during spring, followed by a higher peak the next autumn. The aim of this study was to evaluate whether the incidence of bronchiolitis hospitalization has changed during the last 2 decades in Tampere, Finland.

**Methods:**

In this retrospective register-based study, data on infants <12 months of age hospitalized with bronchiolitis in 2000–2019 were collected from electronic files of Tampere University Hospital and analyzed by monthly incidences. Additionally, data on RSV incidences were collected from the Finnish National Infectious Diseases Register for children <5 years of age and living in the study area. Poisson’s regression analysis was used to evaluate changes in the incidence rates of bronchiolitis.

**Results:**

Of the 1481 infants hospitalized with bronchiolitis, 82.0% had a diagnosis of RSV bronchiolitis. At first, bronchiolitis’ epidemiological pattern followed its typical biannual pattern, then shifted to annual in the middle of the study period, and thereafter occurred biannually again. The highest incidence rate ratios compared to the low-incidence months were between December (22.5), January (25.8) and February (25.5) in 2000–2006, and between February (24.7), March (25.1) and April (21.0) in 2007–2019.

**Conclusions:**

The epidemiological pattern of bronchiolitis changed during the study period; incidence peaks were higher and have shifted toward spring in recent years.

Bronchiolitis is an acute lower respiratory tract infection (LRTI) that begins with symptoms of an upper respiratory infection such as nasal discharge and proceeds to lower respiratory tract symptoms in the following days. These symptoms often include increased effort to breathe, cough, wheezing and fever.^1^ The definition of bronchiolitis varies globally, but generally, in Europe, it is the first LRTI episode with breathing difficulty under the age of 1 year.^2^ Prematurity and congenital heart disease increase the risk for severe bronchiolitis, as well as low birth weight, low social class and tobacco smoke exposure, especially maternal smoking. Bronchiolitis causes hospitalizations of approximately 13.5–24.2/1000 persons/year, but it varies significantly in different areas.^3,4^ Respiratory syncytial virus (RSV) causes approximately 80% of bronchiolitis cases in infants under the age of 6 months, other common etiological agents being rhinoviruses, metapneumoviruses and types 1–3 parainfluenza viruses.^5,6^ Epidemic seasons of bronchiolitis and RSV coincide more than other etiological agents’ incidences, and therefore their epidemics follow a quite similar pattern.^7^

Previous studies have shown that peaks in RSV incidence are associated with cold weather conditions, air humidity and traffic-derived air pollutants such as benzene. Epidemics typically start when the temperature drops and epidemic peaks occur during the coldest months.^1,7,8^ The fact that in temperate regions bronchiolitis’ incidence is clearly seasonal when compared with tropical regions substantiates this theory. The size of epidemic peaks also varies, possibly according to RSV’s genome variation, herd immunity and duration of subtypes A and B.^9^

The epidemiology of bronchiolitis in Nordic countries has historically followed a typical biannual pattern: First, a smaller epidemic occurs during spring, which subsides in summer. During the following autumn, a new epidemic season begins and lasts until the next summer with a higher peak than the earlier epidemic season.^10^ However, based on our clinical experience, it seems that a change has occurred in epidemiology. The difference between the 2 epidemic peaks of RSV has flattened for unknown reasons.^11^ The aim of this study is to find out whether the incidence of bronchiolitis hospitalization has changed during the last 2 decades in Tampere, Finland. A secondary aim was to describe the incidences of RSV and bronchiolitis in Tampere with a special interest to evaluate if these incidence peaks were comparable or not.

## MATERIALS AND METHODS

### Patients

We performed a retrospective register-based study involving patients from Tampere University Hospital’s (Tays) region during 2000–2019. This region is the second largest suburban area in Finland with both urban and rural areas. Tays provides secondary and tertiary levels of care for Pirkanmaa Hospital District, which includes 23 municipalities and a population of over 500,000 by the end of 2019, and is responsible for the district’s pediatric hospital care. The population of infants under the age of 12 months in the region has altered during the data collection period. The number of deliveries in the region has been decreasing from approximately 6000 in 2000 to 4500 in 2019, according to the StatFin database.^12^

In total, 3 different data were collected in this study. The patient data from Tays was collected to answer the primary aim. Information for evaluating the RSV incidence from Finnish National Infectious Diseases Register was collected for the secondary aim. Finally, the weather data of monthly mean temperatures were collected from national weather registers.

First, patient data were collected from the electronic files of Tays. Data includes all patients <12 months of age diagnosed with International Classification of Diseases, 10th Revision (ICD-10 Clinical Modification) codes J21.0 (RSV bronchiolitis) or J21.99 (unspecified bronchiolitis) in Tays’ pediatric ward or emergency room (ER) between January 1, 2000, and December 31, 2019, and thereafter admitted at the pediatric ward or in the intensive care unit. Bronchiolitis diagnoses were mainly clinical, but RSV bronchiolitis was identified based on the ICD-10 code (J21.0). In the ER, viral tests for RSV are usually taken if the child is admitted to the ward and not for every child that was discharged, but some variation in physicians’ practices remains. The data included gender, age on admission, day of admission, length of hospitalization and possible intensive care unit period, possible diagnosis of prematurity and gestational age at birth. Patients were considered premature if they were born before the gestational age of 37 weeks.

Second, the incidence of laboratory-confirmed RSV was evaluated with bronchiolitis incidence. For this purpose, a separate dataset was collected. The number of RSV cases was collected from the Finnish National Infectious Diseases Register, an open database of the National Institute for Health and Welfare in Finland. This database includes information from all Finnish microbiology laboratories. Details from the Tays district’s laboratories only were selected for this study. The monthly number of laboratory-confirmed RSV cases was available for fixed age groups, so the youngest group, including children under 5 years of age, was selected. Unfortunately, data were not available for infants under 12 months only.

Third, weather data of monthly mean temperatures in Tampere in 2000–2019 were collected from the Finnish Meteorological Institute’s website.^13^ The data were used in this study describing whether there was an association between mean temperatures and epidemiological patterns.

The study was approved by the research manager of Tays. Patients were not contacted, so, according to Finnish law, permission from the ethics committee was not required. Data were pseudonymized in such a way that all unnecessary personal data were removed.

## DATA MANAGEMENT AND ANALYSIS

Data were first collected by the hospital technician and exported to Excel from Tays’s electronic files, and information of gestational age was added afterward for a few patients who lacked the information. Statistical analysis was performed using IBM SPSS Statistics for Windows, version 25.0 software (IBM Corp., New York, USA). Categorical variables were described with number and percentages and continuous variables with medians (Md) and lower and upper quartiles (Q_1_ and Q_3_), as the variables were non-normally distributed. The analyses were performed for the total study period and separately for the periods 2000–2006 and 2007–2019.

For the primary aim, the incidences of hospitalized bronchiolitis and of bronchiolitis treated in the pediatric intensive care unit (PICU) were calculated by dividing the monthly number of bronchiolitis or PICU patients by the number of infants under 12 months old living in the area at the end of each year^12^ The length of hospital stay was calculated in days starting from the day of admission being day 0.

Poisson’s regression analysis was used with both year and month as independent variables at the same time in the model to evaluate whether there were changes in the incidence of bronchiolitis, and the results were presented as incidence rate ratios (IRRs) and their 95% confidence intervals (95% CIs). Years were defined as continuous variables, and months were defined as categorical variables. Since the epidemiological patterns seemed to differ by the size and the timing of incidence peaks of bronchiolitis at the beginning and the end of the study period, the entire data for the 2 decades were analyzed, and additionally, divided into 2 sections to evaluate the IRRs’ and epidemiological pattern’s changes between them. Since the year 2006 had the lowest incidence of bronchiolitis in the period including that and the nearest years, the periods 2000–2006 and 2007–2019 were analyzed separately. July, August and September were the months with the lowest bronchiolitis incidence; thus, all other months’ incidences were compared to them. Results with *P*-values less than 0.05 were considered statistically significant.

For the secondary aim, the RSV incidence was calculated by dividing the monthly number of confirmed RSV cases (children <5 years of age) by the number of children in the same age group living in the area,^12^ and this information was reported with the annual incidence of bronchiolitis. Additionally, the monthly mean temperatures were presented together with the bronchiolitis and RSV incidences, but not statistically analyzed.

## RESULTS

During the study period, 1481 patients were hospitalized due to bronchiolitis in the Tays region (Table 1). The majority was boys (57.9%), and their median age on the day of admission was 84 days. Within the 1219 patients for whom information of gestational age at birth was available, the majority were born full-term (84.7%). Most common (82%) diagnosis was RSV-bronchiolitis (ICD-10 J21.0), whereas all other possible bronchiolitis-causing viruses comprised the rest of the cases (ICD-10 J21.99, unspecified bronchiolitis). The median length of hospitalization was 4 days in 2000–2006 compared to 2 days in 2007–2019, and altogether 99 patients (6.7%) were treated in the PICU during the study period (5.4% in 2000–2006 compared to 7.2% in 2007–2019) (Table 1).

**TABLE 1.** Description of Patients (n = 1481) with Bronchiolitis at Age Under 12 Months and Treated in the Tampere University Hospital, Finland, during 2000–2019

Variable	2000–2019 n (%)	2000–2006 n (%)	2007–2019 n (%)
Gender			
Male	857 (57.9)	228 (55.9)	629 (58.6)
Female	624 (42.1)	180 (44.1)	444 (41.4)
Diagnosis			
RSV bronchiolitis	1214 (82.0)	408 (100)	806 (75.1)
Unspecified bronchiolitis	267 (18.0)	-	267 (24.9)
Prematurity, gestational age <37 weeks			
Yes	187 (12.6)	40 (9.8)	147 (13.7)
No	1032 (69.7)	150 (36.8)	882 (82.2)
Missing information	262 (17.7)	218 (53.4)	44 (4.1)
PICU			
Yes	99 (6.7)	22 (5.4)	77 (7.2)
No	1382 (93.3)	386 (94.6)	996 (92.8)
	Md (Q_1_;Q_3_)		
Age (days)	84 (45.5;153)	85 (46.3;151.8)	84 (45;153.5)
Length of stay (days)	3 (1;5)	4 (2;6)	2 (1;5)
Gestational age (weeks)	39 (38;40)	39 (37;40)	39 (38;40)

Md indicates median; PICU, pediatric intensive care unit; Q1, lower quartile; Q3, upper quartile; RSV, respiratory syncytial virus.

**FIGURE 1.**
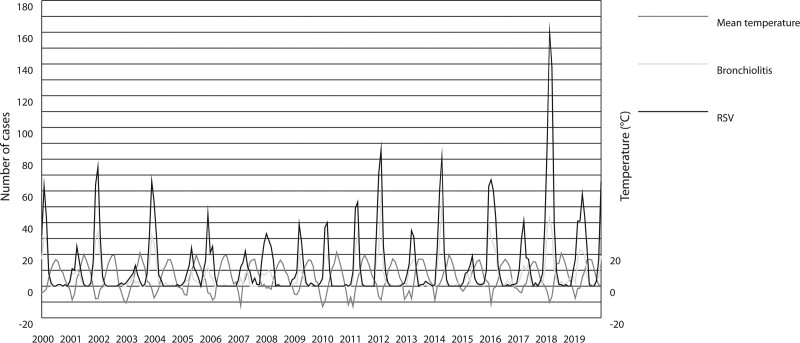
Monthly number of infants <12 months of age with bronchiolitis and children <5 years of age with laboratory-confirmed RSV in the area of Tampere University Hospital, Finland, and their connection with monthly mean temperatures in Tampere.

In the beginning of the study period, bronchiolitis epidemiology followed the same biannual pattern with unequal peaks as before. Figure 1 shows that in the middle of the study period (2008–2012), the epidemiology altered toward a more annual pattern with equal peaks each year. Thereafter, the pattern reverted to its original, biannual form. Monthly mean temperatures followed a regular pattern throughout the study period, and the onset of bronchiolitis’ and RSV’s epidemiological peak usually began when temperatures dropped (Fig. 1).

**FIGURE 2.**
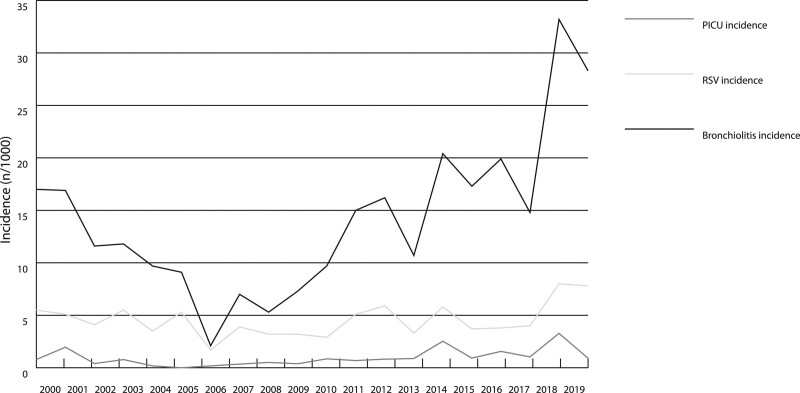
Incidences bronchiolitis, laboratory-confirmed RSV and pediatric intensive care unit (PICU) admissions for bronchiolitis in the area of Tampere University Hospital, Finland.

The bronchiolitis incidence was comparable to RSV incidence for 2 decades, as their peaks always appeared at the same time. The highest monthly number (n = 59) of hospitalization due to bronchiolitis was in Feb. 2012. (Fig. 1). Bronchiolitis incidence was clearly higher, but the patterns of epidemics were similar, despite the fact that bronchiolitis’ incidence had increased more than RSV’s incidence (Fig. 2). Bronchiolitis’ and RSV’s incidences were at their lowest at the same time, as the epidemiological model had changed to the annual form, and the incidence increased at the same time as the biannual pattern returned.

During the early years, in 2000–2006, the incidence trend was decreasing (IRR for year 0.8, 95% CI: 0.8–0.9) and after that, in 2007–2019, it trended upward (IRR for year 1.1, 95% CI: 1.1–1.15) (Table 2). In the overall data collection period, there was a significant increase in bronchiolitis incidence (IRR for year 1.1, 95% CI: 1.0–1.1) (Table 2).

**TABLE 2.** The Incidence Rate Ratios of Bronchiolitis Reported For The Whole Study Period 2000–2019 and Separately for The Years 2000–2006 and 2007–2019

	2000–2019 IRR (95% CI)^*^	*P*-value^†^	2000–2006 IRR (95% CI)	*P*-value^†^	2007–2019 IRR (95% CI)	*P*-value^†^
Years (as continuous)^‡^	1.1 (1.0–1.1)	<0.001	0.8 (0.8–0.9)	<0.001	1.1 (1.1–1.15)	<0.001
Months^§^						
January	16.2 (11.4–23.0)	<0.001	25.8 (13.4–49.7)	<0.001	12.7 (8.3–19.3)	<0.001
February	24.9 (17.7–35.0)	<0.001	25.5 (13.2–49.1)	<0.001	24.7 (16.5–36.8)	<0.001
March	22.2 (15.8–31.3)	<0.001	14.4 (7.3–28.5)	<0.001	25.1 (16.8–37.4)	<0.001
April	17.5 (12.4–24.8)	<0.001	8.1 (3.9–16.7)	<0.001	21.0 (14.0–31.4)	<0.001
May	10.6 (7.4–15.3	<0.001	6.0 (2.8–12.8)	<0.001	12.3 (8.1–18.8)	<0.001
June	3.1 (2.0–4.8)	<0.001	3.6 (1.6–8.3)	0.003	2.9 (1.7–5.0)	<0.001
July, August, September	1 (reference)		1 (reference)		1 (reference)	
October	2.3 (1.4–3.7)	0.001	2.7 (1.1–6.6)	0.031	2.1 (1.2–3.8)	0.013
November	5.6 (3.8–8.3)	<0.001	10.8 (5.4–21.8)	<0.001	3.7 (2.2–6.1)	<0.001
December	14.8 (10.4–21.0)	<0.001	22.5 (11.6–43.5)	<0.001	11.9 (7.8–18.1)	<0.001

^*^IRR = incidence rate ratios; 95% CI = confidence interval of 95%.

^†^A *P*-value for IRR, considered statistically significant if < 0.05.

^‡^Years were defined as continuous variables.

^§^Reference months July, August and September, which had the lowest bronchiolitis incidences during the study period. Months were defined as categorical variables.

The peak incidence shifted during the study period: during the early years, in 2000–2006, the highest IRRs were in December, January and February, and after that (in 2007–2019), February, March and April had the highest IRRs, when compared to July, August and September (Table 2). During the overall data collection period, in 2000–2019, February and March had the highest IRR compared to the reference months (IRR >20.0) (Table 2).

## DISCUSSION

In this register-based study, we evaluated whether infant bronchiolitis’ epidemiological pattern has altered during the last 2 decades, 2000–2019, in the Tays region, Finland. The main result was that epidemiological patterns had shifted over the study period, expressing both annual and biannual patterns. In addition, we found an overall increase in bronchiolitis’ IRR over the whole study period, even though during 2000–2006 IRR had been decreasing. There was a link between the incidence and the change in epidemiological pattern since the IRR was at its lowest during the occurrence of the annual pattern.

In line with the present study, an earlier Finnish register study, in 1995–2018, recognized that the epidemiological pattern of RSV had changed to an annual pattern in all age groups after 2006, and the higher epidemiological peaks existed in 2016 and 2018.^11^ In this present study, we found that the original biannual epidemiological pattern of bronchiolitis and RSV had returned in our study population after 2012 with a higher IRR than before. Both of these studies show that the epidemiological patterns of bronchiolitis’ and RSV’s incidences have altered during the last 2 decades, for reasons that remain unclear.

Previous studies have shown an increase in the trend of RSV- and non-RSV-bronchiolitis’ hospitalization rates. In a broad retrospective register study in Brazil that included 263,679 infants under the age of 1 year, the annual hospitalization rate increased from 8.5/1000 in 2008 to 12.7/1000 in 2015.^14^ Annual bronchiolitis hospitalizations steadily increased from 6.6/1000 to 46.1/1000 between 1979 and 2011 in a register study including 468,138 hospital admissions of infants under the age of 1 year in the UK.^3^ In contrast, a decrease in bronchiolitis hospitalizations from 17.9 to 13.5 per 1000 person/years was found in a serial, cross-sectional study of 490,650 children under the age of 2 years in the United States in 2000–2016.^4^ In our study, we discovered that bronchiolitis’ IRRs first showed a declining trend (IRR 0.8) until after 2006 when the IRR started increasing (IRR 1.1). Over the entire study period, the trend was slightly upward. The proportion of ICU admissions among all bronchiolitis hospitalizations increased from 11.7% in 2010 to 24.5% in 2019 in a retrospective, cross-sectional study of 203,859 children under the age of 2 years in the United States.^15^ In our study population, the overall proportion of ICU admissions was lower (6.7%), without a clear temporal trend.

A study in Italy, in which 723 previously healthy full-term infants under the age of 1 year were enrolled, examined the association between the timing of bronchiolitis’ epidemic peaks and possible affecting factors in the period 2004–2014 and found that the incidence peaks occurred in the winter months of January and February, earlier than they expected.^7^ A shift in the peak incidence was also demonstrated in our study, although the shift occurred toward spring. In the first period (2000-2006) of our study, the months with the highest IRR were December, January, and February, whereas, in the later period, they were February, March, and April. The timing of RSV’s epidemiological peaks was studied globally in a study based on country-specific medical databases and RSV surveillances of 27 countries worldwide. Clear peaks occurred in temperate countries, whereas in tropical regions, RSV was present steadily throughout the year without remarkable peaks.^16^ In line with this observation, our findings show clear peaks in the incidence of bronchiolitis and RSV, as Finland is a temperate country.

In the prospective observational study mentioned above, 14 different respiratory viruses were analyzed, and the effect of climate factors on bronchiolitis incidence was studied. A statistically significant inverse correlation was found between temperature and RSV bronchiolitis.^8^ In line with the Italian study, a multicenter study of 3876 children 2–12 months old in Australia and New Zealand in 2009–2011 found a statistically significant correlation between cold temperatures, wind speed and bronchiolitis incidence. No correlation was found between relative humidity and rainfall. Viral detection was not conducted in this study.^17^ Although detailed weather analysis was beyond the scope of our study, our findings are in line with these studies, as the highest incidence of bronchiolitis occurred during the colder winter months. Thus, changes in climate factors could affect the timing of bronchiolitis incidence peaks. The mechanism behind this association remains unclear.

As the number of bronchiolitis admissions increases, the overall need for healthcare services, together with the costs associated with bronchiolitis treatment, also increases. Previous studies from Finland demonstrated that costs of bronchiolitis hospitalization were between €1,900 and €15,600 depending if the infant was treated in the ward or in the PICU.^18,19^ Additionally, costs are even higher when viewed from the perspective of families or society.^19,20^ The increase in the IRR of bronchiolitis demonstrated in our study predicts a greater burden for healthcare services; therefore, it is important to recognize the epidemiological pattern to prepare suitable strategies to reduce excess costs of this disease.

The major strength of this study was the high-quality real-world patient data. As Tays is the only hospital providing pediatric inpatient care in the area, we know that all infants who were hospitalized due to a clinical diagnosis of bronchiolitis were identified and included into the data. The Finnish National Infectious Diseases Register is a systemically maintained database that provides reliable data of confirmed RSV cases in Finland, including the Tays region. Our study also has some limitations. Unfortunately, we had no data on potential viral etiologies of bronchiolitis other than RSV, and we could not differentiate the subtypes of RSV. Moreover, we have only limited details collected from the patients, and therefore, for example, the comorbidities could not be analyzed. Obviously, in this kind of real-world data some patients have comorbidities, which may influence both the severity of bronchiolitis and the length of stay in hospital. However, it is presumptive that the comorbidities did not affect the temporal changes in the bronchiolitis incidence. Although the length of our study period is a strength the total number of patients remained quite low due to the small population living in the Tays region.

In conclusion, a changed pattern of bronchiolitis incidence was found during the last 2 decades; the epidemic peak was higher and occurred later in the spring, toward the end of the study period.

## References

[1] Florin TA Plint AC Zorc JJ. Viral bronchiolitis. Lancet. 2017;389:211–224. 27549684 10.1016/S0140-6736(16)30951-5 PMC6765220

[2] Jartti T Smits HH Bønnelykke K.; EAACI Task Force on Clinical Practice Recommendations on Preschool Wheeze. Bronchiolitis needs a revisit: distinguishing between virus entities and their treatments. Allergy. 2019;74:40–52. 30276826 10.1111/all.13624 PMC6587559

[3] Green CA Yeates D Goldacre A. Admission to hospital for bronchiolitis in England: trends over five decades, geographical variation and association with perinatal characteristics and subsequent asthma. Arch Dis Child. 2016;101:140–146. 26342094 10.1136/archdischild-2015-308723 PMC4752648

[4] Fujiogi M Goto T Yasunaga H. Trends in bronchiolitis hospitalizations in the United States: 2000-2016. Pediatrics. 2019;144:e20192614. 31699829 10.1542/peds.2019-2614 PMC6889950

[5] Midulla F Scagnolari C Bonci E. Respiratory syncytial virus, human bocavirus and rhinovirus bronchiolitis in infants. Arch Dis Child. 2010;95:35–41. 19822538 10.1136/adc.2008.153361

[6] Miller EK Gebretsadik T Carroll KN. Viral etiologies of infant bronchiolitis, croup and upper respiratory illness during 4 consecutive years. Pediatr Infect Dis J. 2013;32:950–955. 23694832 10.1097/INF.0b013e31829b7e43 PMC3880140

[7] Cangiano G Nenna R Frassanito A. Bronchiolitis: analysis of 10 consecutive epidemic seasons. Pediatr Pulmonol. 2016;51:1330–1335. 27228123 10.1002/ppul.23476 PMC7167938

[8] Nenna R Evangelisti M Frassanito A. Respiratory syncytial virus bronchiolitis, weather conditions and air pollution in an Italian urban area: an observational study. Environ Res. 2017;158:188–193. 28647513 10.1016/j.envres.2017.06.014 PMC7125886

[9] Hogan AB Anderssen RS Davis S. Time series analysis of RSV and bronchiolitis seasonality in temperate and tropical Western Australia. Epidemics. 2016;16:49–55. 27294794 10.1016/j.epidem.2016.05.001

[10] Waris M. Pattern of respiratory syncytial virus epidemics in Finland: two-year cycles with alternating prevalence of groups A and B. J Infect Dis. 1991;163:464–469. 1995719 10.1093/infdis/163.3.464

[11] Renko M Tapiainen T. Change in respiratory syncytial virus seasonality in Finland. Acta Paediatr. 2020;109:202–203. 31441534 10.1111/apa.14983

[12] StatFin online service [Internet]. Statistics Finland. Available at: https://www.stat.fi/tup/statfin/index.html. Accessed March 23, 2021.

[13] Finnish Meteorological Institute [Internet]. Cited 2022 June 22. Available at: https://en.ilmatieteenlaitos.fi/. Accessed October 31, 2021.

[14] Tumba K Comaru T Machado C. Temporal trend of hospitalizations for acute bronchiolitis in infants under one year of age in Brazil between 2008 and 2015. Rev Paul Pediatr. 2020;38:e2018120. 31778406 10.1590/1984-0462/2020/38/2018120 PMC6909255

[15] Pelletier JH Au AK Fuhrman D. Trends in bronchiolitis ICU admissions and ventilation practices: 2010-2019. Pediatrics. 2021;147:e2020039115. 33972381 10.1542/peds.2020-039115 PMC8785748

[16] Obando-Pacheco P Justicia-Grande AJ Rivero-Calle I. Respiratory syncytial virus seasonality: a global overview. J Infect Dis. 2018;217:1356–1364. 29390105 10.1093/infdis/jiy056

[17] Hoeppner T Borland M Babl FE.; Paediatric Research in Emergency Departments International Collaborative (PREDICT). Influence of weather on incidence of bronchiolitis in Australia and New Zealand. J Paediatr Child Health. 2017;53:1000–1006. 28727197 10.1111/jpc.13614

[18] Heikkilä P Forma L Korppi M. Hospitalisation costs for infant bronchiolitis are up to 20 times higher if intensive care is needed. Acta Paediatr. 2015;104:269–273. 25431309 10.1111/apa.12881

[19] Mäntynen E Palmu S Heikkilä P. Families’ costs form a considerable part of total costs in bronchiolitis care. Health Sci Rep. 2022;5:e593. 35509378 10.1002/hsr2.593 PMC9059178

[20] Miedema CJ Kors AW Tjon A Ten WE. Medical consumption and socioeconomic effects of infection with respiratory syncytial virus in The Netherlands. Pediatr Infect Dis J. 2001;20:160–163. 11224834 10.1097/00006454-200102000-00008

